# Conductance Studies on Complex Formation between c-Methylcalix[4]resorcinarene and Titanium (III) in Acetonitrile-H_2_O Binary Solutions

**DOI:** 10.3390/molecules181012041

**Published:** 2013-09-27

**Authors:** Majid Rezayi, Yatimah Alias, Mahnaz M. Abdi, Kasra Saeedfar, Naghmeh Saadati

**Affiliations:** 1 Food Quality and Safety Research Department, Food Science and Technology Research Institute, ACECR Mashhad Branch, 91775-1376 Mashhad, Iran; 2 Chemistry Department, Faculty of Science, University Malaya, 50603 Kuala Lumpur, Malaysia; 3 Department of Chemistry, Faculty of Sciences, Universiti Putra Malaysia, 43400 Serdang, Selangor D.E., Malaysia; 4 School of Chemical Sciences and Food Technology, Faculty of Science and Technology, University Kebangsaan Malaysia, 43600 Bangi, Selangor D.E., Malaysia; 5 Chemistry Department, Faculty of Science, K. N., Toosi University of Technology, 15418-49611 Tehran, Iran

**Keywords:** conductance data, calixresorcinarenes, titanium(III), c-methylcalix[4]- resorcinarene, acetonitrile (AN)-water (H_2_O), binary mixtures

## Abstract

Calixresorcinarenes have proved to be unique molecules for molecular recognition via hydrogen bonding, hydrophobic and ionic interactions with suitable substrates such as cations. The study of the interactions involved in the complexation of different cations with calixresorcinarenes in solvent mixtures is important for a better understanding of the mechanism of biological transport, molecular recognition, and other analytical applications. This article summarizes different aspects of the complexes of the Ti^3+^ metal cation with c-methylcalix[4]resorcinarene (CMCR) as studied by conductometry in acetonitrile (AN)–water (H_2_O) binary mixtures at different temperatures. Conductance data show that the metal cation/ligand (ML) stoichiometry of the complexes in solution is 1:1 in all cases. Non-linear behaviour was observed for the variation of log*K_f_* of the complexes *vs*. the composition of the binary solvent mixtures. Selectivity of CMCR for the Ti^3+^ cation is sensitive to solvent composition; in some cases and at certain compositions of the mixed solvent systems, the selectivity order is changed. Values of thermodynamic parameters (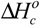,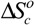) for formation of the CMCR–Ti^3+^ complexes in AN–H_2_O binary systems were obtained from the temperature dependence of stability constants, and the results show that the thermodynamics of complexation reactions are affected by the nature and composition of the mixed solvents.

## 1. Introduction

**Figure 1:**
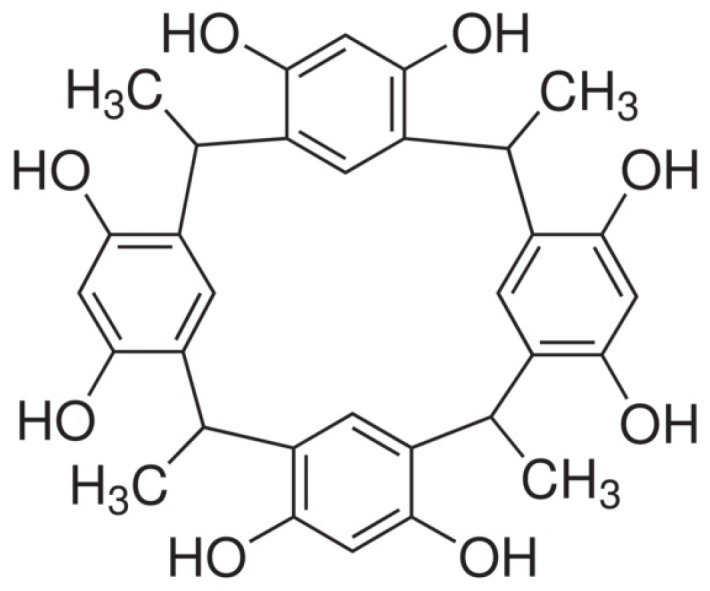
Molecular structure of C-methylcalix[4]resorcinarene (CMCR).

Investigations with calixarenes and calixresorcinarenes have demonstrated the versatility of these molecules in supermolecular host–guest chemistry [1]. c-Methylcalix[4]resorcinarene (CMCR, Figure 1) is an excellent building block for the construction of a variety of hydrogen-bonded networks, thanks to the large number of hydroxyl groups along its rim, allowing it to act as a hydrogen bond donor in producing extended structures or binding ligands to give an extended cavity capable of taking up large guests, such as ferrocene [2]. Coppens *et al.* showed that 4,4′-bipyridine is capable of forming a variety of inclusion compounds with CMCR, with the CMCR molecules in any of four conformations: crown, boat, chair and scoop [3]. However, in all reported open boat conformation CMCR molecules, the CMCR either forms a discrete complex with an inorganic ion, or each CMCR molecule hydrogen bonds directly to at least one adjacent CMCR, resulting in ‘brick wall’ motifs, while the bipyridine serves to link these ‘walls’ together. Shivanyuk *et al.* have reported the ability of CMCR to adopt the boat conformation without forming the brick walls described by Coppens in his investigations of bipyridyl-type ligands [4]. In this instance, chloride ions served to extend the boat-shaped cavity to allow accommodation of the triethylammonium ion. Use of such inorganic ions presents an interesting alternative route to produce extended resorcinarene structures. Recently, calixarenes, which are macrocyclic products of phenol–formaldehyde condensations, have been shown to be amenable to chemical modifications at the upper or lower rim to yield new macromolecules and act as effective ionophores that can selectively bind a range of metals [5]. In addition to calixarenes, structurally related resorcinarenes, which are the macrocyclic products of resorcinol with aldehydes (except formaldehyde), form complexes with both cations and polar organic molecules. Until now, only a few reports on the use of these resorcin[n]arenes for extraction of metals have appeared. Several groups have studied the novel resorcin[4]arenes, and their complexation with alkali metal ions, especially with potassium ions [6,7]. Due to this fact, many researchers have examined a wide range of applications for these macrocyclic compounds in different areas, such as the construction of ion selective electrodes (ISEs) [8], separation of metal cations [9], as stationary phases in chromatography columns [10], in the design of chemical sensors [11], recognition of isomers [12], and chemical analysis [13].

In recent years, this branch of chemistry has seen a lot of advances. Many new novel compounds with high selectivities for ion or molecule separation, transport and catalytic purposes have been developed by scientists active in different areas of chemistry. Diverse methods, such as UV–Vis spectroscopy, polarography, potentiometry, and conductometery have been applied to investigation of the complexometric reactions [14,15].

Conductivity measurements are useful tools for investigating complexation, due to their precision, simplicity, and low cost [16]. In this work, complexations between CMCR and titanium (Ti^3+^) cation in water (H_2_O), acetonitrile (AN), and their binary mixture solutions at different temperatures have been studied using the conductometric method. The goal of the present investigation was to study the effect of solvent properties and the composition of the binary mixed solvents on the stoichiometry and the selectivity of complexes formed between ligands and cations in various solvent systems.

## 2. Results and Discussion

The conductometric method was used in the thermodynamic study of complexation reactions between the CMCR ligand with the Ti(OH)(OH_2_)_5_^2+^ cation in acetonitrile–water (AN–H_2_O) binary mixtures at different temperatures. It was also used to evaluate the effect of the composition of acetonitrile–water binary solutions on the nature of titanium cations. The results could be useful for applications such as the fabrication of ISEs based on macrocyclic ionophores for determining titanium ions in real samples [17,18,19].

**Figure 2:**
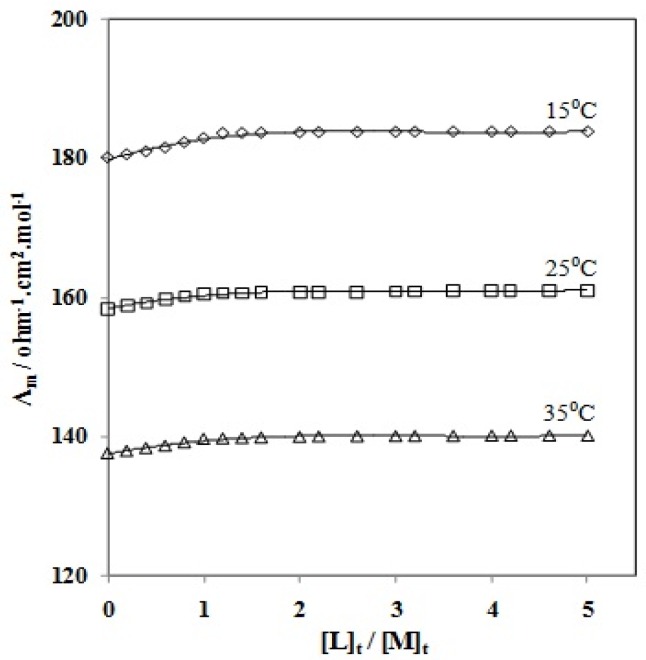
Molar conductance (S cm^2^.mol^−1^) *vs*. [CMCR]/[Ti(OH)(OH_2_)_5_^2+^] mole ratio plots in pure AN at various temperatures.

**Figure 3:**
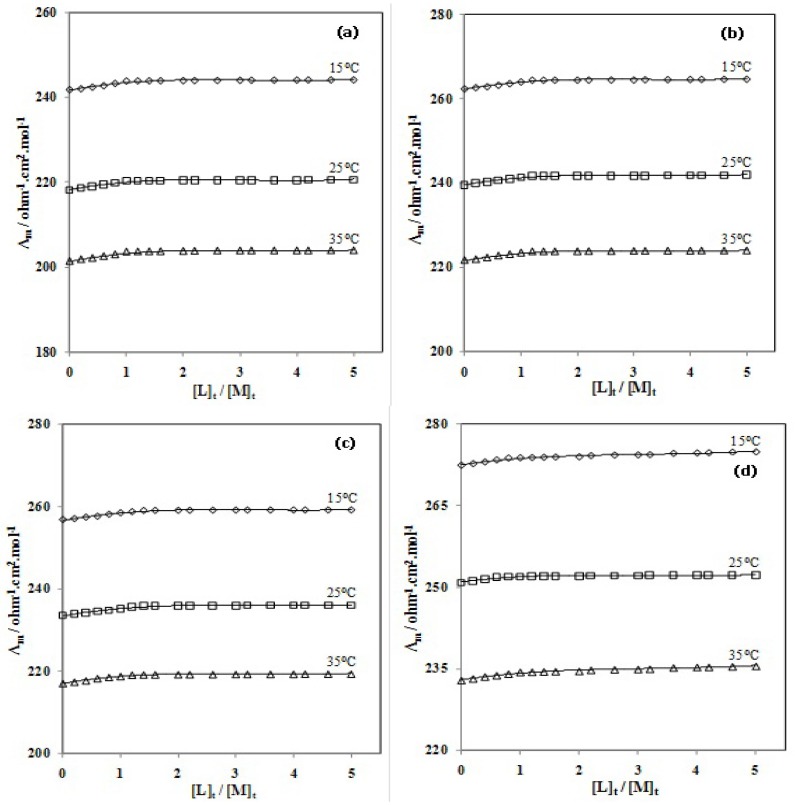
Molar conductance (S cm^2^.mol^−1^) *vs*. [CMCR]/[Ti(OH)(OH_2_)_5_^2+^] mole ratio plots in AN–H_2_O binary mixture (mol% H_2_O = (**a**):42.05, (**b**):65.91, (**c**):81.32, (**d**):92.07) at various temperatures.

Changes in molar conductivity (Λ_m_) *vs*. the molar ligand-to-ion ratio of CMCR to the Ti(OH)(OH_2_)_5_^2+^ cation ([*L*]_t_/[*M*]_t_, where [*L*]_t_ is the total concentration of the ligand and [*M*]_t_ is the total concentration of the titanium (III) ion) for the complexation reactions were measured in pure AN and in AN–H_2_O binary systems at three different temperatures. A result in pure H_2_O was not obtained, due to the low solubility of CMCR. Five typical series of the resulting molar conductance *vs*. CMCR/Ti(OH)(OH_2_)_5_^2+^ molar ratio plots in pure AN and the AN–H_2_O binary mixtures (mol% H_2_O = 42.05, 65.91, 81.32 and 92.07) are shown in Figure 2 and Figure 3.

As can be seen, the addition of the CMCR ligand to titanium cation solution in pure AN and in AN–H_2_O binary mixtures at different temperatures increased the molar conductivity (Λ_m_) values as the CMCR/Ti(OH)(OH_2_)_5_^2+^ molar ratio increased, which indicates that CMCR–Ti(OH)(OH_2_)_5_^2+^ complexes in these systems have higher mobility than the free solvated Ti(OH)(OH_2_)_5_^2+^ cation. The slope of the corresponding mole ratio plots changes at the point where the ligand to cation mole ratio corresponds to about a 1:1 complex between the CMCR ligand and the Ti(OH)(OH_2_)_5_^2+^ cation. The main goal of the conductometric study was to obtain stability constant complexes between ligands and ions. Formation constants of all the CMCR-Ti(OH)(OH_2_)_5_^2+^ complexes in different solvent mixtures at various temperatures, obtained by computer fitting of the molar conductance–mole ratio data, are listed in Table 1.

**Table 1:** Formation constants for different CMCR-Ti(OH)(OH_2_)_5_^2+^ complexes in AN–H_2_O binary mixtures at various temperatures.

Medium	Log K_f_ ± SD ^a^		
	15 °C	25 °C	35 °C
Pure AN	3.59 ± 0.06	3.62 ± 0.08	3.65 ± 0.20
57.95% AN-42.05% H_2_O	3.29 ± 0.03	3.30 ± 0.04	3.37 ± 0.18
34.09% AN-65.91% H_2_O	2.86 ± 0.05	3.04 ± 0.07	3.06 ± 0.07
18.68% AN-81.32% H_2_O	2.97 ± 0.13	2.86 ± 0.30	3.02 ± 0.10
7.93% AN-92.07% H_2_O	2.62 ± 0.16	2.83 ± 0.17	2.87 ± 0.21
Pure H_2_O	c	c	c

^a^ SD = Standard deviation; ^b^ the composition of each solvent system is expressed in mole% of each solvent;

^c^ ion or ligand salt is insoluble.

From Table 1, it is obvious that the nature of the solvent has a fundamental effect on the stability of the resulting complexes. In most cases, the stability constant of the complexes increases with the temperature in most solvent systems, which is evidence for an endothermic complexation reaction between ligands and ions in the solutions. There are many reasons for discussing the effectiveness of solvents on the stability constant of complexes, owing to the fact that this factor changes from one solvent system to another.

As shown in Table 1, the stability of the complex increases with the decreasing solvating power of the solvent mixtures (H_2_O), as expressed by the Gutman donor number. Water is a solvent with a high solvating ability (DN = 18) and hence solvates the ion more strongly than acetonitrile (DN = 14.1). As a result, replacement of the solvent molecules around the cation by the calixarenes would be energetically expensive. This effect is observed for all different mol% of the AN–H_2_O mixtures. For instance, the logK_f_ values for (CMCR–Ti(OH)(OH_2_)_5_)^2+^ in 100% AN, 57.95% AN–H_2_O, 34.09% AN–H_2_O, 18.68% AN–H_2_O and 7.93% AN–H_2_O are 3.62, 3.30, 3.04, 2.86, and 2.83 in 25 °C, respectively.

**Figure 4:**
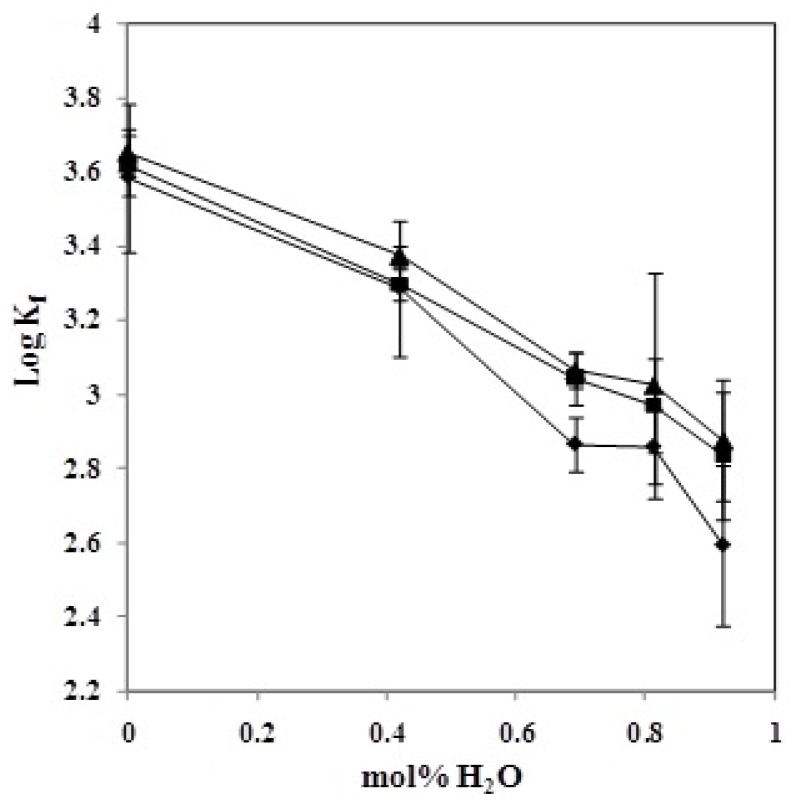
Variation of the stability constant (log K_f_) of (CMCR-Ti(OH)(OH_2_)_5_) ^2+^ complex with the composition of AN–H_2_O binary systems at different temperatures: (♦15 °C, ■ 25 °C, ▲ 35 °C).

As shown in Figure 4, changes in the stability constant (log*K_f_*) of the (CMCR–Ti(OH)(OH_2_)_5_)^2+^ complex *vs*. the composition of the AN–H_2_O binary system at various temperatures are not linear. This behaviour is probably due to solvent–solvent interactions that change the structure of the solvent mixtures and therefore change the solvation properties of the titanium cation, the CMCR, and the resulting complex. In addition, preferential solvation of the cation and ligand, as well as the characteristics of its changes with the composition of the mixed solvents and temperature, may be other reasons for this kind of behaviour. The interactions between some binary mixed solvents have been studied. For example, mixing dimethylformamide with acetonitrile induces the mutual destruction of the dipolar structures of these dipolar aprotic liquids and releases the free dipoles. As a result, strong dipolar interaction between acetonitrile and water molecules is expected. Similar behaviour was observed in other cases.

**Figure 5:**
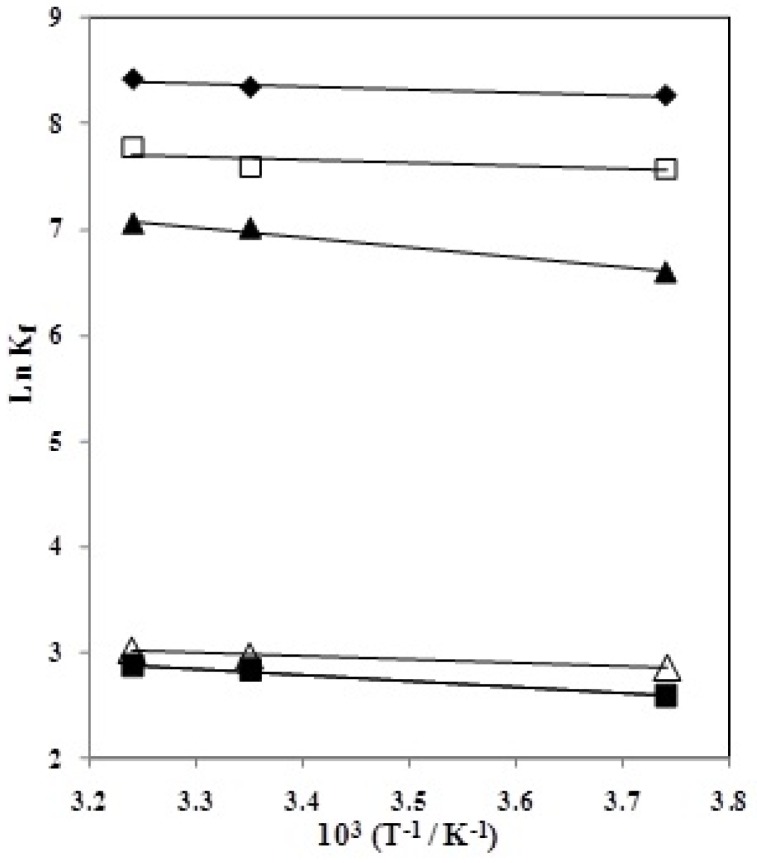
Van’t Hoff plots for the 1:1 complexation of Ti(OH)(OH_2_)_5_)^2+^ cation with CMCR in AN–H_2_O binary systems: (mol% AN: ♦ 100, □ 57.95, ▲ 34.09, ∆ 18.68, ■ 7.93).

The two fundamental equations: ∆*G* = −*RT* ln *K_f_* and ∆*G* = ∆*H* − *T*∆*S* were used to compare the contributions of enthalpy and entropy towards the stability of the CMCR–Ti(OH)(OH_2_)_5_^2+^ complexes. The enthalpy contribution can be obtained experimentally by using van’t Hoff plots [20]. In order to have a better understanding of the thermodynamics of complexation reactions of Ti(OH)(OH_2_)_5_)^2+^ cations with CMCR, it is useful to investigate the enthalpic and entropic contributions to these reactions. The 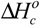 and 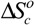 of the complexation reactions in different AN–H_2_O mixtures were evaluated from the temperature dependence of the formation constants by applying a linear least-squares analysis according to the Van’t Hoff equation. The Van’t Hoff plots of ln *Kf* vs. 1/*T* are shown in Figure 5. The enthalpies and entropies of complexation were determined in the usual manner from the slopes and intercepts of the plots, respectively, and the results are listed in Table 2.

**Table 2:** Thermodynamic parameters for different CMCR-Ti(OH)(OH_2_)_5_^2+^ complexes in various AN–H_2_O binary mixtures.

Medium	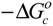 ± SD^a^ kJ/mol	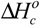 ± SD^a^ kJ/mol	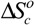 ± SD^a^ J/mol.^⁰^K
Pure AN	20.66 ± 0.47	5.54 ± 0.12	87.90 ± 1.52
57.95% AN-42.05% H_2_O	18.82 ± 0.23	7.20 ± 3.41	87.27 ± 11.41
34.09% AN-65.91% H_2_O	17.37 ± 0.41	16.66 ± 7.25	114.18 ± 24.33
18.68% AN-81.32% H_2_O	16.95 ± 0.73	13.90 ± 2.36	103.47 ± 7.52
7.93% AN-92.07% H_2_O	16.18 ± 0.97	23.80 ± 9.05	134.10 ± 30.19

^a^ SD = Standard deviation; ^b^ The composition of each solvent system is expressed in mole% of each solvent.

The calculated thermodynamic parameters (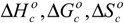) at 25 °C for CMCR-Ti(OH)(OH_2_)_5_^2+^ complexes in pure AN and AN–H_2_O binary systems are listed in Table 2. The given experimental values of standard enthalpy (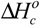) and standard entropy (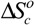) show that, in most cases, the complexes are enthalpy destabilized, but entropy stabilized. Therefore, the entropy of the complexation reactions is the principal driving force for the formation of these complexes in most solvent systems. The experimental results show that, in most cases, the change in standard enthalpy for the complexation reaction between ligand and ion is negligible; therefore, it seems that the complexation processes in most of the solvent systems are probably athermic.

## 3. Experimental

### 3.1. Materials

The host CMCR was used as purchased from Fluka (Seelze, Germany). Reagent grade 15% titanium (III) chloride, and dried acetonitrile (max. 0.005%H_2_O) (all from Merck, Darmstadt, Germany) were of the highest purity available and were used as received. All the AN–H_2_O mixtures used were prepared by weight. Deionized bi-distilled water was used throughout the experiment. Conductance measurements were carried out with a digital HI 255 conductivity meter (Hanna Instruments, Ann Arbor, MI, USA). A dip-type conductivity cell made of platinum black was used. The cell constant at the different temperatures used was determined by conductivity measurements of a 0.1000 M solution of analytical-grade KCl (Merck) in deionized water. In all measurements, the cell was thermostatted at the desired temperature ±0.01 °C using a 630D thermostat-circulator water bath (Protech, San Antonio, Texas, USA).

### 3.2. Methodology

In a typical experiment, 25 mL of the desired metal ion (5.0 × 10^−5^ M) were placed in the titration cell, thermostatted to the desired temperature, and the conductance of solution was measured. Then, a known amount of a concentrated CMCR solution (2.5 × 10^−3^ M) was added in a stepwise manner using a calibrated micropipette. The conductance of the solution was measured after each addition. The ligand solution was added continually until the desired ligand to cation mole ratio was achieved. The formation constants, *K_f_*, and the molar conductances, Λ_m_, of the resulting 1:1 complexes between CMCR and the titanium (III) cation used, in different AN–H_2_O mixtures and at various temperatures, were calculated by fitting the observed molar conductance, Λ_obs_, at varying [CMCR]/[Ti^3+^] mole ratios to a previously derived equation that expresses the Λ_obs_ as a function of the free and complexed titanium (III) ions. A nonlinear least squares curve fitting using the GENPLOT computer program was applied to evaluate the formation constant and the limiting molar conductance of the resulting 1:1 complexes [21]. In the present work, all of the measured solutions were adjusted in pH range 1–3, and the dominant titanium species were in the form of Ti(OH)(OH_2_)_5_^2+^ [17,18,22].

## 4. Conclusions

The stability constants of the complex formation between the CMCR and the titanium (III) cations were measured based on the conductometric method in various mole percentages of AN–H_2_O media systems at different temperatures. The results obtained showed that, in the case of the CMCR–Ti(OH)(OH_2_)_5_^2+^ complex, the stability constant of the complex decreased with an increasing percentage of water.

The negative quantities of standard Gibbs free energy and the positive values of entropy indicate spontaneous complexation reactions between the CMCR ligand and the titanium (III) cations. CMCR forms a 1:1 complex with the Ti(OH)(OH_2_)_5_^2+^ cation at all compositions of the AN–H_2_O binary solution. The average value of the stability constant (ln *K_f_*) for the CMCR–Ti(OH)(OH_2_)_5_^2+^ complexes at 25 °C was 3.13. In conclusion, based on the results obtained, the CMCR ligand can be used as a desired ionophore to detect the titanium (III) cation in the field of the chemical sensor.
